# Sex differences in the gut microbiome and related metabolites: role in cardiometabolic disease

**DOI:** 10.1080/19490976.2026.2721752

**Published:** 2026-08-26

**Authors:** Yi Wang, En Cheng, Brandilyn A. Peters-Samuelson

**Affiliations:** a Department of Epidemiology and Population Health, Albert Einstein College of Medicine, Bronx, NY, USA

**Keywords:** sex differences, gut microbiome, gut microbiota-related metabolites, sex steroid hormones, cardiometabolic disease

## Abstract

Sex differences in major cardiometabolic diseases (CMD), such as type 2 diabetes, metabolic dysfunction-associated steatotic liver disease, and cardiovascular disease, have been increasingly recognized across the life course. During the reproductive stage, women generally have a more favorable cardiometabolic risk profile than men, but their risk of experiencing CMD significantly increases after menopause. Emerging evidence suggests that sexually dimorphic gut microbiota and gut microbiota-related metabolites (GMRMs) may contribute to such sex disparities. However, existing findings are heterogeneous and underlying mechanisms remain incompletely understood. In this review, we synthesize the evidence examining sex differences in gut microbial diversity, overall composition, taxa abundances, and levels of GMRMs across key life stages, including pre-puberty, adolescence, and different phases of adulthood (with emphasis on pre- and post-menopausal periods). We summarize potential biological mechanisms underlying sexual dimorphism in the gut microbiome and GMRMs, emphasizing the central role of sex steroids, along with contributions from immune function and other host and environmental factors. We further integrate evidence linking sexually dimorphic microbial taxa (e.g., *Akkermansia muciniphila*, *Eubacterium*, *Ruminococcus*, and other Firmicutes taxa) and GMRMs (e.g., microbiota-derived short-chain fatty acids, secondary bile acids, and trimethylamine N-oxide) to key cardiometabolic pathways involving inflammation, glucose and lipid metabolism, and vascular function. Finally, we identify critical knowledge gaps and emphasize the need for future large longitudinal studies that would integrate repeated measurements of the gut microbiome, untargeted metabolomics, and sex steroid hormones across the life course. Such approaches are essential to clarify biological pathways and inform sex-specific microbiome-based interventions for CMD prevention and management.

## Introduction

Cardiometabolic diseases (CMD), such as type 2 diabetes (T2D), metabolic dysfunction-associated steatotic liver disease (MASLD), and cardiovascular disease (CVD), are major causes of mortality worldwide.^1^ However, men and women differ remarkably in pathological manifestations for disease onset, progression, prognosis, and disease risk factors, with women generally exhibiting a more favorable cardiometabolic profile.^2^ ^,^ ^3^ For example, while the lifetime risks of CVD in men and women are similar, the manifestations vary across stages of the life course: men have a higher incidence of CVD at a younger age than women,^4^ while women have higher morbidity and mortality rates after menopause due to declining estrogen levels.^5^ ^-^ ^7^ Regarding metabolic conditions, while women generally have a higher prevalence of obesity than men,^8^ particularly severe obesity,^9^ men have a higher risk of developing severe conditions (such as T2D^10^ and MASLD^11^). Despite these differences, prevention, diagnostics, and treatment guidelines are applied equally to both sexes. In addition, underrepresentation of women in clinical trials^3^ and female animals in basic science research^12^ may further impede the implementation of sex-specific precision medicine. In all, there is a substantial knowledge gap in sex-specific mechanisms and clinical management for CMD.

Sex differences in pathophysiological mechanisms, clinical features, and responses to CMD therapies can be attributed to a multitude of physiological, environmental, and sociocultural factors,^3^ ^,^ ^13^ ^,^ ^14^ such as hormonal effects, body composition, immune function, host genetics, epigenetic modification, lifestyle, and stress.^3^ ^,^ ^15^ Women and men have different metabolic homeostasis in response to endogenous and exogenous factors which possibly influence the manifestations of CMD.^16^ In recent years, gut microbiome dysbiosis and host-microbe interactions have been increasingly recognized as contributors to the observed sex disparities in CMD incidence, through effects on gut barrier dysfunction, inflammatory pathways activation, and microbial metabolites production.^2^ ^,^ ^17^ Given the modifiable nature of gut microbiota (e.g., through dietary and probiotic interventions), understanding the roles of sexually dimorphic gut microbiome and microbial metabolites in CMD can inform personalized prevention and treatment for women and men. Thus, we summarize up-to-date knowledge of sex differences in gut microbiota and microbial metabolites and their underlying mechanisms contributing to CMD. This narrative review primarily synthesizes evidence from human studies, while incorporating relevant mechanistic findings from animal studies where appropriate. The literature search strategy for human studies is summarized in Supplementary Table 1. Our review may provide a theoretical and practical basis to optimize sex-specific interventions for CMD management.

## Gut microbiome

The human gut microbiome is defined as the entire complex microbial community of approximately 10^13^ to 10^14^ microorganisms, including bacteria, archaea, fungi, and viruses, among which bacteria represent the dominant fraction.^18^ ^,^ ^19^ They inhabit the gastrointestinal tract and the colon contains the highest microbial density. These colonizing gut microbes can encode > 3 million genes, 150-fold more than those present in the human genome.^20^ The microbiome displays considerable taxonomic and functional diversity,^21^ as well as large interindividual variability,^22^ which can be influenced by both endogenous (e.g., sex, biological age, host genotype, immune function)^23^ ^-^ ^25^ and exogenous (e.g., antibiotic use, diet, smoking, stress) factors.^26^ The gut microbiota carries out many important physiological functions, such as aiding food digestion and nutrient absorption, maintaining metabolic homeostasis, and regulating immune function.^27^ ^,^ ^28^ Specifically, gut microbiota can contribute to the metabolism of essential dietary components (e.g., fiber, amino acids) and host-derived endogenous compounds (e.g., primary bile acids, hormones). Over the past two decades, advances in next-generation sequencing technology and the development of state-of-the-art bioinformatics tools have enabled characterization of the gut microbiome at the resolution of single microbial genomes, allowing strain-level and functional analyses, and thus expanding the study of microbial communities to an unprecedented degree.^29^ ^,^ ^30^ The application of these technologies and tools in epidemiological studies has rapidly advanced our understanding of the unique roles of gut microbiota in human health and disease. Bacteria, as the dominant and most extensively studied members of the gut microbiota, are therefore the primary focus of this review. The term “gut microbiome” in this review refers to bacteria studied in stool samples, though we also summarize emerging evidence on the gut mycobiome (fungi), virome (viruses), and archaeome (archaea).

The gut microbiota plays critical roles in metabolic, homeostatic, and immunological functions that maintain human health.^31^ Gut microbiota dysbiosis is characterized by reduced bacterial diversity, loss of beneficial taxa, and enrichment of detrimental taxa, which can lead to chronic inflammation and oxidative stress, thereby disrupting host pathophysiological processes.^32^ A growing body of studies has linked the gut microbiome to a wide range of chronic diseases and conditions, such as obesity, T2D, MASLD, CVD, inflammatory bowel disease, colorectal cancer, and depression.^33^ ^,^ ^34^ Research in this field is motivated by the strong translational potential to utilize the gut microbiota as both biomarker and modifiable target for disease prevention and therapy.^35^ The gut microbiome is shaped by multiple host and environmental factors; notably, sex is among the top 10 contributors accounting for interindividual variation.^26^

## Gut microbiota-related metabolites (GMRMs)

Gut microbiota can modulate host metabolism and immune function by producing a variety of metabolites, which have gained increasing attention in recent studies.^36^ These small molecules, produced by gut microbes or derived from microbial metabolism of dietary components, are important for intestinal homeostasis by regulating food digestion, energy balance, gut barrier integrity, and immune responses.^25^ ^,^ ^37^ ^,^ ^38^ Beyond the intestine, GMRMs can affect multiple organs through the systemic circulation, such as adipose tissue metabolism (adipose tissue), glucose homeostasis (pancreas), lipid metabolism (liver), and cognitive function (brain).^39^ ^,^ ^40^ Beneficial taxa produce antioxidative and anti-inflammatory metabolites that can regulate metabolic homeostasis.^25^ ^,^ ^41^ In contrast, overrepresentation of detrimental bacteria is associated with the accumulation of pro-inflammatory metabolites or metabolites that disrupt metabolic homeostasis, contributing to the development of metabolic disorders.^25^ Certain GMRMs have been well-studied. For example,

- Lipopolysaccharide (LPS; also known as endotoxin) is a unique cell wall component of Gram-negative bacteria, and translocation of LPS into circulation can induce inflammatory responses.^42^ ^,^ ^43^
- Short-chain fatty acids (SCFAs) are generated by bacterial fermentation of non-digestible carbohydrates and are known to exert broad beneficial effects by regulating inflammation, immune response, adipose tissue metabolism, energy expenditure, and glucose and lipid homeostasis, thereby contributing to anti-inflammatory, immunoregulatory, anti-obesity, anti-diabetic, cardioprotective, and hepatoprotective properties.^40^ ^,^ ^44^
- Trimethylamine N-oxide (TMAO) is produced from dietary nutrients (e.g., choline and L-carnitine) and can increase the risk of metabolic and cardiovascular disorders.^36^ ^,^ ^45^
- Indole and its derivatives are produced by gut microbial tryptophan metabolism and are involved in maintenance of intestinal homeostasis and barrier function by regulating the balance of pro- and anti-inflammatory cytokine expression.^46^ ^,^ ^47^
- Secondary bile acids (e.g., lithocholic acid and deoxycholic acid) are derived from primary bile acids through gut microbial deconjugation and have been associated with inflammation, carcinogenesis, and metabolic diseases.^48^

High-resolution mass spectrometry technology (e.g., liquid chromatography-tandem mass spectrometry and gas chromatography-mass spectrometry) enables more comprehensive assessment of gut microbiota-derived metabolites in fecal, blood or urine samples.^49^ ^,^ ^50^ GMRMs provide a common and critical mechanistic link between gut microbiota and physiological and pathological processes, such as energy expenditure,^51^ substrate oxidation,^52^ gut dysbiosis,^53^ inflammation,^53^ and immune system regulation,^54^ offering insights into host-microbe interactions in human health and disease. Importantly, accumulating evidence from advanced research demonstrates that specific GMRMs exert direct biological effects with therapeutic potential. For example, urolithin A, which is produced by gut bacteria from dietary polyphenols, has demonstrated potential anti-aging effects.^36^ The pleiotropic actions of GMRMs demonstrate their importance as both biomarkers and targets for future translational research and clinical therapy.

## Sex differences in gut microbiome and GMRMs

### Gut microbiome (bacteria)

Despite growing interest in the role of the gut microbiome in health and disease, sex differences in gut microbiome remain understudied. Over the past two decades, a growing number of human studies have compared gut microbiome composition between women and men from different populations (Table 1). Overall, findings across these studies are heterogeneous and largely inconclusive, potentially due to differences in methodology and study populations. Major sources of heterogeneity include:

**Table 1.** Summary of human studies on sex differences in the gut microbiome across the life course.

Authors, publication year	Location	Sample size	Age (y)	Study method	Findings related to sex differences (females vs. males)
**Children (<18 y)**					
Yatsunenko et al. [55]	Malawi, Venezuela, USA	For sex comparison with monozygotic and dizygotic twin pairs, 16 for neonatal and infant pairs, and 50 for teenaged pairs from the U.S.	Neonatal and infant (1–12 months) and teenage (13–17 y)	Gut microbiome: stool, 16S	Monozygotic and dizygotic twin pairs from the US Neonatal and infant: no difference in β-diversity Teenage: significant difference in β-diversity
Hollister et al. [56]	USA	19 girls and 18 boys	Total mean ± SD: 9.5 ± 1.4	Gut microbiome: stool, 16S	No difference in α- or β-diversity
Yuan et al. [57]	China	Pre-puberty: 14 girls and 28 boys; Puberty: 26 girls and 21 boys	Mean ± SD Pre-puberty: Girls: 8.0 ± 1.2 Boys: 8.5 ± 1.8 Puberty: Girls: 10.7 ± 0.9 Boys: 11.4 ± 1.3	Mucosa-associated microbiome: 16S	Pre-puberty No difference in α- or β-diversity Taxa abundance: ↑ Family *Lactobacillaceae*, S24_7, Genus *Lactococcus* ↓ Phylum TM7, Class TM7_3 Puberty α-diversity: no difference β-diversity: significant difference Taxa abundance: ↑ Family *Odoribacteraceae*, *Rikenellaceae*, *Porphyromonadaceae*, *Bacteroidaceae*, Genus *Clostridium, Holdemania, Anaerotruncus, Bilophila, Oscillospira* ↓ Order Lactobacillales, Actinomycetales, Coriobacteriales, Class Coriobacteriia, Family Micrococcaceae, Genus *Megamonas, Rothia, Lactococcus, Adlercreutzia*
**Adulthood**					
Mueller et al. [58]	France, Germany, Italy, Sweden	132 females and 98 males (*n* = 85 aged 20–50 and *n* = 145 aged > 60 y)	Mean (range) Females: 60 (24–94) Males: 59 (25–100)	Gut microbiome: stool, 14 group- and species-specific 16S rRNA-targeted oligonucleotide probes	Taxa abundance: ↓ *Bacteroides-Prevotella group*
Aguirre de Cárcer et al. [59]	Australia	5 females and 5 males	Mean ± SD Females: 57.5 ± 7.7 Males: 54.3 ± 9.9	Gut microbiome: stool, qPCR	Taxa abundance: ↑ *Streptococcus*, *Veillonella*, *Mannheimia*, *Ruminococcus spp*. ↓ *Faecalibacterium prausnitzii, Bacteroides, Clostridium, Prevotella, Enterococcus, Bifidobacterium spp.*
Feng et al. [60]	China	62 females and 46 males	Median (IQR) Females: 46.5 (42.0–50.0) Males: 46.0 (43.0–50.3)	Gut microbiome: stool, qPCR	Taxa abundance: ↑ *Faecalibacterium prausnitzii*
Ding and Schloss [61]	USA	151 females and 149 males	Total mean: 26	Gut microbiome: stool, 16S	Taxa abundance: ↑ *Bacteroides* ↓ *Prevotella*
Dominianni et al. [62]	USA	31 females and 51 males	Mean ± SD Females: 59.2 ± 12.7 Males: 58.0 ± 13.6	Gut microbiome: stool, 16S	β-diversity: significant difference Taxa abundance: ↓ Bacteroidetes
Oki et al. [63]	Japan	325 females and 191 males	Total mean ± SD: 52.4 ± 13.4	Gut microbiome: stool, 16S	α-diversity: ↑ β-diversity: no difference Taxa abundance:
					↑ *Christensenellaceae, Dehalobacteriaceae, Mogibacteriaceae, Oxalobacteraceae, Verrucomicrobiaceae, Victivallaceae* ↓ *Actinomycetaceae, Fusobacteriaceae, Gemellaceae, Paraprevotellaceae, Pseudomonadaceae,* S24-7, *Succinivibrionaceae*
Haro et al. [64]	Spain	36 post-menopausal females and 39 males (age-matched)	Mean ± SD Females: 60.3 ± 1.4 Males: 61.2 ± 1.3	Gut microbiome: stool, 16S	No difference in α- or β-diversity Taxa abundance (regardless of BMI): ↑ *Bilophila, Bacteroides caccae* ↓ *Veillonella, Methanobrevibacter, Bacteroides plebeius, Coprococcus catus*
Borgo et al. [65]	Italy	20 females and 20 males	Total mean ± SD: 29.3 ± 8.9	Gut microbiome: stools (lumen-associated microbiota, LAM), sigmoid brush (mucosa-associated microbiota, MAM), 16S	MAM α-diversity: ↑ β-diversity: significant difference Taxa abundance: ↑ *Actinobacteria, Lactobacillales, Streptococcaceae* *, Bifidobacterium adolescentis,* ↓ Veillonellaceae, unclassified Clostridia*, Gemmiger formicilis, Phascolarctobacterium faecium* LAM No difference in microbial features
Gao et al. [66]	China	292 females and 259 males	Mean ± SD Females: 36.5 ± 16.3 Males: 38.3 ± 16.2	Gut microbiome: stool, 16S	α-diversity: ↑ β-diversity: no significant difference Taxa abundance: ↑ *Ruminococcus* (Regardless of the BMI)
Santos-Marcos et al. [67]	Spain	17 pre-menopausal females, 19 males (age-matched), 20 post- menopausal females and 20 males (age-matched)	Mean ± SD Pre-menopausal females: 46.1 ± 0.8 Males (age-matched to pre-menopausal women): 46.6 ± 0.6 Post-menopausal: 55.9 ± 0.7 Males (age-matched to post-menopausal women): 56.2 ± 0.7	Gut microbiome: stool, 16S	Pre-menopausal females vs. males (age-matched) α-diversity: no significant difference β-diversity: significant difference Taxa abundance: ↑ *Prevotella*, Actinobacteria, *Desulfovibrionaceae*, Coriobacteriia, *Coriobacteriales*, etc. ↓ Veillonellaceae Post-menopausal females vs. males (age-matched) α-diversity: no significant difference β-diversity: significant difference Taxa abundance: ↑ *Bacteroides plebeius*, Lactobacillales ↓ Odoribacteraceae, *Parabacteroides distasonis*, *Odoribacter, Parabacteroides,* Bacteroidaceae, etc.
Mahnic et al. [68]	Slovenia	117 females and 69 males	Total mean (range): 45.2 (18.0–85.0)	Gut microbiome: stool, 16S	α-diversity: ↑ β-diversity: significant difference Taxa abundance: ↑ *Akkermansia,* Ruminococcaceae spp.*, Alistipes* spp. ↓ *Blautia, Roseburia*, *Lachnospiraceae*, *Butyricicoccus*.
Takagi et al. [69]	Japan	139 females and 138 males	Total range: 20–89	Gut microbiome: stool, 16S	α-diversity: no difference β-diversity: significant difference Taxa abundance: ↑ *Bifidobacterium, Ruminococcus, Akkermansia, Unclassified* [Barnesiellaceae]*, Eggerthella, Anaerotruncus,* Unclassified ML615J-28*, rc4-4, Clostridium3, Christensenella, Anaerofustis* ↓ *Prevotella, Megamonas, Fusobacterium, Megasphaera, Eubacterium, Mitsuokella, Paraprevotella, Desulfovibrio, Actinomyces,* Unclassified Gemellaceae*, Moryella, Urobacterium, Abiotrophia*
Sinha et al. [70] *	Netherlands	661 females and 474 males	Total mean ± SD: 45.0 ± 13.6	Gut microbiome: stool, shotgun metagenomic sequencing	α-diversity: ↑ β-diversity: significant difference Taxa abundance: ↑ *Akkermansia muciniphila, Lachnospiraceae bacterium 7 1 58FAA, Subdoligranulum unclassified, Eggerthella unclassified, Eubacterium eligens, Gordonibacter pamelaeae, Lachnospiraceae bacterium 3 1 57FAA CT1, Alistipes onderdonkii, Lachnospiraceae bacterium 5 1 63FAA, Bilophila unclassified* ↓ *Collinsella aerofaciens, Bifidobacterium longum* Functional pathways (MetaCyc): 43 differential metabolic pathways, with 32 enriched (e.g., TCA cycle III (animals)) and 11 depleted (e.g., sucrose degradation IV (sucrose phosphorylase)) in women (see Supplementary Table 1 in the original article)
Shin et al. [71]	South Korea	26 females and 31 males	Mean ± SD Females: 46.2 ± 2.3 Males: 37.5 ± 1.9	Gut microbiome: stool, 16S	No difference in α-, β-diversity, or taxa abundance.
de la Cuesta-Zuluaga et al. [72]	UK, USA, China, Colombia	Five cohorts (AGP, U.S., AGP, U.S. antibiotic consumers, AGP, U.K., Chinese, Colombian cohorts) Young adults: 2047 females and 1703 males Middle-aged adults: 3134 females and 2497 males	Young adults (ages 20–45 y) Middle-aged adults (ages 46–69 y)	Gut microbiome: stool, 16S	α-diversity: ↑ U.S., U.K., Colombian cohorts Null: Chinese cohort
Mayneris-Perxachs et al. [73] *	Spain	44 pre-menopausal females, 45 post-menopausal females, 42 males	Mean (IQR) Pre-menopausal females: 41.9 (34.4–48.4) Post-menopausal females: 58.6 (55.4, 59.6) Males: 46.1 (39.2, 54.3)	Gut microbiome: stool, shotgun metagenomic sequencing	α-diversity: no difference among pre- and post-menopausal females and males Pre-menopausal females vs. males β-diversity: significant difference Taxa abundance: 273 differential species ↑ Most species belong to Firmicutes, such as *Ruminococcus sp. CAG:379, Clostridiales bacterium VE202-26, Clostridium neonatale, Enterococcus* ↓ Most species belong to Bacteroidetes, such as *Prevotella sp. S7-1-8, Bacteroides sp. CAG:661, Prevotella buccalis* Functional pathways (KEGG pathway): 32 differential metabolic pathways, with 18 enriched (e.g., steroid biosynthesis and degradation) and 14 depleted (e.g., Nicotinate and nicotinamide metabolism) in women (see Figure 2a in the original article) Post-menopausal females vs. males β-diversity: no difference Taxa abundance: 90 differential species ↑ Most species belong to Firmicutes, such as *Anaerovibrio sp. JC8, Streptococcus thermophilus, Clostridia bacterium UC5.1 + 1C12* ↓ Most species belong to Firmicutes and Bacteroidetes, such as *Roseburia sp. CAG:10041_57*, *Roseburia sp. CAG:100*, *Bacteroides sp. CAG:927*, *Clostridium sp. CAG:590* Functional pathways (KEGG pathway): ↑ Arginine biosynthesis and metabolism
Zhang et al. [74] *	China	Discovery: 1191 females and 1147 males in the Pinggu (PG) Cohort in China Validation: three population data from China (five published studies, 461 females and 415 males), Israel (adults without diabetes, 522 females and 353 males), and the Netherlands (LifeLines DEEP, 660 females and 473 males)	Median (range) PG: 48 (26–76) Chinese (five public data): 48 (19–86) Israeli: 40 (18–70) Dutch: 45 (18–81)	Gut microbiome: stool, shotgun metagenomic sequencing	α-diversity: ↑ β-diversity: significant difference Taxa abundance: ↑ *Alistipes shahii, Alistipes sp*. *HGB5*, *Akkermansia muciniphila*, *Eggerthella lenta, Blautia hydrogenotrophica, Pseudoflavonifractor capillosus, Anaerotruncus colihominis, Clostridium symbiosum, Clostridiales bacterium 1_7_47FAA, Erysipelotrichaceae bacterium 3_1_53* Functional pathways (KEGG orthology [KO]): ↑ single-strand selective monofunctional uracil DNA glycosylase (K10800), butyrate biosynthesis (K00248 and K01034), β-glucuronidase (K01195) ↓ bile acid transporter (K03453) (These findings were consistent in four cohorts; see Supplementary Table 9 in the original article)
Peters et al. [75] *	USA	295 pre-menopausal females, 1027 post-menopausal females, 258 younger males, and 720 older males	Mean ± SD Pre-menopausal females: 40.1 ± 8.8 Younger males: 42.7 ± 8.7 Post-menopausal females: 61.0 ± 7.2 Older males: 61.2 ± 7.1	Gut microbiome: stool, shotgun metagenomic sequencing	Pre-menopausal females vs. younger males α-diversity: ↑ β-diversity: significant difference Taxa abundance: ↑ *Escherichia coli-Shigella spp., Oscillibacter sp. strain KLE1745, Akkermansia muciniphila, Clostridium lactatifermentans, Escherichia coli, Parabacteroides johnsonii, Veillonella seminalis* ↓ *Bacteroides sp. strain Ga6A1, Prevotella marshii, Sutterella wadsworthensis* Functional pathways (KEGG functional modules): ↑ Type IV secretion system, alpha-hemolysin/cyclolysin transport system ↓ Sulfate transport system Post-menopausal females vs. older males α-diversity: not available β-diversity: significant difference Taxa abundance: ↑ *Oscillibacter sp. strain KLE1745, Akkermansia muciniphila, Clostridium lactatifermentans* ↓ *Bacteroides sp. strain Ga6A1, Prevotella marshii, Sutterella wadsworthensis* Functional pathways (KEGG functional modules): NA Regarding the taxa abundance, only the species that differed between post- and pre-menopausal women in this study were further shown the comparisons in the above-mentioned groups. The volcano plot (Figure 2a) in the original article suggests a greater number of differential species, but the full list of these species was not provided.
Vriend et al. [76]	Netherlands	2693 females and 2473 males	Mean (IQR) Females: 50 (42, 58) Males: 52 (42, 58)	Gut microbiome: stool, 16S	α-diversity: ↑ β-diversity: significant difference Taxa abundance:
					↑ *Blautia faecis, Odoribacter splanchinicus, Alistipes putredenis, Alistipes obesi, Bilophila wadsworthia, Eubacterium eligens group spp., Clostridia spp., Streptococcus spp., Akkermansia muciniphila, Christensenellaceae spp., UCG−005 spp., Lachnospiraceae spp.* ↓ *Prevotella_9 copri*, *Enterorhabdus spp., Ruminococcus torques group spp., Sutterella massiliensis/stercoricanis/wadsworthensis, Colidextribacter spp., Lachnoclostridium spp., Phascolarctobacterium succinatutens, Slackia isoflavoniconvertens*
Garcia‑Fernandez et al. [17]	Spain	87 females and 242 males without CVD	Mean ± SE Females: 59.6 ± 0.9 Males: 59.2 ± 0.6	Gut microbiome: stool, 16S	α-diversity: no difference β-diversity: significant difference Taxa abundance: ↑ Firmicutes, Verrucomicrobiota, Verrucomicrobiae, Akkermansia, Verrucomicrobiales, Akkermansiaceae, Oscillospiraceae, *Christensenellaceae_R-7_group*, Christensenellales, Christensenellaceae ↓ *Collinsella*, Coriobacteriaceae, Bacteroidales, Bacteroidota, Bacteroidia
Shalmon et al. [77]	Israel	9 females and 13 males	Total mean ± SD: 43 ± 6.5	Gut microbiome: stool, 16S	α-diversity: no difference β-diversity: no difference Taxa abundance: ↑ *Methanosphaera*, *Lactobacillus* ↓ *Enterococcus*
**All ages**					
Lay et al. [78]	France, Denmark, Germany, Netherlands, United Kingdom	Total subjects: 91. France (*n* = 21), Denmark (*n* = 20), Germany (*n* = 20), Netherlands (*n* = 20), United Kingdom (n*=*10)	Range: 7–52	Gut microbiome: stool, fluorescent in situ hybridization using 18 phylogenetic probes	According to the principal component analysis, there is no significant grouping of samples with respect to gender in the entire samples or sub-samples in specific country
Li et al. [79]	China	4 females and 3 males (four-generation Chinese family)	Range Females: 1.5–95 Males: 18–55	Gut microbiome: stool, 16S Samples were collected twice, 30 d apart	β-diversity: significant difference Taxa abundance: ↓ Clostridia, Bacteroidetes, Proteobacteria, *Bacteroides thetaiotaomicron (using Bacteroides spp.* group-specific DGGE profiling*)*
Singh and Manning [80]	USA	200 individuals with diarrhea (93 females and 104 males), 75 healthy family members (33 females and 37 males)	Age group: 0–10 (*n* = 91) 11–18 (*n* = 32) 19–49 (*n* = 84) 50–69 (*n* = 45) ≥70 (*n* = 12)	Gut microbiome: stool, 16S	β-diversity: significant difference Taxa abundance: Healthy individuals ↑ *Dialister*, *Lactobacillus*, unclassified *Lactobacillaceae*, unclassified Comamonadaceae, *Comamonas* ↓ *Paraprevotella* Individuals with diarrhea ↑ Bacteroidaceae predominated among the 43 differentially abundant features ↓ Enterobacteriaceae predominated among the 11 differentially abundant features
Li et al. [81] *	China	90 females and 95 males	Range: 14–70	Gut microbiome: stool, shotgun metagenomic sequencing	α-diversity: no difference β-diversity: no difference Taxa abundance: ↑ *Bacteroides (B. cellulosilyticus, B. ovatus, B. stercoris, B. thetaiotaomicron, B. uniformis, Bacteroides sp. 3_1_23, Bacteroides sp. 3_1_33FAA), Parabacteroides (P. merdae), Clostridium (C. bolteae, C. symbiosum, Clostridium sp. HGF2), Akkermansia (A. muciniphila)* Functional pathways (KEGG orthology): 439 gender-associated KOs ↑ KOs involved in carbohydrate metabolism, energy metabolism, enzyme families, and glycan biosynthesis and metabolism ↓ KOs involved in nucleotide metabolism and metabolism of cofactors and vitamins
Koliada et al. [82]	Ukraine	1515 females and 786 males	Total range: 0–60+	Gut microbiome: stool, qRT-PCR	↑ Firmicutes to Bacteroidetes (F/B) ratio Taxa abundance: Phylum-level ↑ Firmicutes, Actinobacteria ↓ Bacteroidetes Genus and species-level: no difference

Studies are ordered by year of publication within each category. Arrows (↑, ↓) indicate taxa with increased or decreased abundance in females compared with males, respectively. Taxa shown in *italics* denote genus- or species-level classifications. *For studies using shotgun metagenomic sequencing, sex-associated microbial functional pathways are also presented. Abbreviations: DGGE, denaturing gradient gel electrophoresis; IQR, interquartile range; SD, standard deviation; SE, standard error; qPCR, quantitative polymerase chain reaction; qRT-PCR, quantitative reverse transcription polymerase chain reaction.

- Age (all ages spanning from infants and adolescents to adults)
- Study population location
- Microbiome profiling approaches (e.g., qRT-PCR, 16S rRNA gene sequencing, or shotgun metagenomic sequencing)
- Biospecimen type (luminal, mucosal, or fecal samples)
- Sample sizes (ranging from 7 to 9381 subjects)
- Health conditions (e.g., general population, patients with gastrointestinal disease or other diseases)

Sexually dimorphic patterns in fat distribution and energy and nutritional requirements across the lifespan may also influence the observed sex differences in gut microbial features.^83^ Many studies demonstrated that sexual dimorphism in the gut microbiome was age-dependent,^67^ ^,^ ^73^ ^,^ ^75^ and findings therefore should be interpreted within a life-course framework. Women and men undergo distinct physiological transitions across the life course. Puberty and menopause are two critical life-stage transitions accompanied by profound hormonal and metabolic alterations. We summarize the existing studies by life stage in Table 1. Because some studies included participants spanning multiple life stages, we categorized these studies as children (<18 y), adults (≥18 y), and all ages in the table, and further discussed the findings by detailed life stages.

**Pre-puberty (neonatal and infant) and adolescence**. To date, only three studies^55^ ^-^ ^57^ have exclusively studied children and supported the importance of gut microbiome in pubertal maturation. Specifically, a multi-country study by Yatsunenko et al. ^55^ reported no sex differences in β-diversity during the neonatal and infant periods. Similarly, smaller studies in the U.S.^56^ and China^57^found no sex differences in α- or β-diversity in pre-pubertal children. In contrast, puberty appears to be a critical transition point. Yuan et al. ^57^ reported significant sex differences in β-diversity among adolescents, accompanied by marked shifts in taxonomic composition. During puberty, girls showed enrichment of taxa such as Odoribacteraceae, Bacteroidaceae, *Clostridium*, and *Oscillospira*, whereas boys had higher abundances of Lactobacillales, *Megamonas*, and *Lactococcus*. A greater number of sex-associated taxa were detected in pubertal children compared with pre-pubertal children,^57^ whereas microbial diversity did not differ by sex regardless of pubertal status.^56^ ^,^ ^57^ These findings might suggest that hormonal changes during puberty coincide with the emergence of sex-specific microbial species, rather than sex per se being a determinant in early life (i.e., from birth to puberty). Since these studies were limited by small sample sizes (*N* < 100) within each developmental stage, future longitudinal studies with larger sample sizes are warranted to characterize the dynamic and sex-specific trajectories of the gut microbiome among children. However, based on current evidence, sex differences in the gut microbiome may be minimal before puberty and become more evident after puberty.

**Young, middle, and older adulthood.** Generally, sex differences in overall gut microbiome composition were observed, with women showing greater microbial diversity and higher Firmicutes/Bacteroidetes (F/B) ratio compared with men,^84^ ^-^ ^86^ particularly in relatively large human studies (*N* > 1000).^70^ ^,^ ^72^ ^,^ ^74-76^ However, the specific differential taxa involved vary across studies. Specifically, in a recent multi-ethnic study of a Dutch population (The Healthy Life in an Urban Setting [HELIUS]; 2693 females and 2473 males [mean age: 50 vs. 52 y]) using 16S rRNA sequencing data,^76^ women had higher abundances of *Akkermansia muciniphila, Christensenellaceae spp., Lachnospiraceae spp., Eubacterium eligens group spp.,* and lower abundances of *Prevotella_9 copri* and *Ruminococcus torques group spp.* compared to men. In a subset of this study (*N* = 259) with metagenomic sequencing data, several sex-associated functional pathways were identified including vitamin B6 biosynthesis (more abundant in women) and stachyose degradation pathways (more abundant in men). Both pathways were correlated with the abundance of *A. muciniphila*, consistent with previous findings from an animal study.^87^ In another Dutch study (LifeLines-DEEP; 661 females and 474 males, mean age: 45 y) using metagenomic sequencing data,^70^ 12 microbial species (e.g., *A. muciniphila, Lachnospiraceae bacterium, E. eligens,* and *Bifidobacterium longum*) and 43 metabolic pathways (e.g., sulfate reduction I, assimilatory, superpathway of sulfate assimilation and cysteine biosynthesis, chondroitin sulfate degradation I [bacterial]) differed by sex. However, after adjustment for diet, lifestyle, and medications, only *A. muciniphila* remained significant, for which women had a higher abundance than men.

Sexual dimorphism of the gut microbiome varied within different stages of adulthood. de la Cuesta-Zuluaga et al. ^72^ found that sex differences in the gut microbiome were more pronounced in younger adults (20–45 y) than in middle-aged adults (46–69 y), with women having higher α-diversity than men, which were observed in the US, UK, and Colombian cohorts though not in a Chinese cohort. Another multi-cohort study^74^ discovered and validated 10 sex-associated species across diverse populations (four cohorts from China, Israel and the Netherlands), including higher abundances of *A. muciniphila, Eggerthella lenta* and several SCFA-producing species (e.g., *Blautia hydrogenotrophica, Anaerotruncus colihominis, Pseudoflavonifractor capillosus)* in women compared with men, which have been implicated in beneficial effects on host metabolic health. At the functional level, women showed enrichment of genes involved in single-strand selective monofunctional uracil DNA glycosylase (K10800), butyrate biosynthesis (K00248 and K01034), and β-glucuronidase (K01195), but depletion of the bile acid transporter (K03453). It is worth noting that sex differences in microbial diversity (higher in women than men), overall composition, and taxonomic profiles were more pronounced in younger adults (26 < age ≤ 50 y) than in middle-aged and older adults (50 < age ≤ 76 y). Furthermore, sex-dependent microbial differences were not observed in pre-pubertal Dutch children, but were evident in younger Chinese, Israeli and Dutch adults and gradually attenuated after age 50. Menopausal status has also been shown to modify sex differences in the gut microbiome during adulthood. Peters et al. ^75^ found that the influence of sex on overall gut microbiome composition and species abundance was significantly larger in younger adults (pre-menopausal women vs. younger men) than in older adults (post-menopausal women vs. older men) in a U.S. Hispanic/Latino population. This study further revealed enrichment of the type IV secretion system and the alpha-hemolysin/cyclolysin transport system, and depletion of the sulfate transport system, in pre-menopausal women compared with younger men; whereas no significant differences in microbial functional modules were detected between post-menopausal women and older men. Similar findings were reported by two smaller studies from Spain,^67^ ^,^ ^73^ which showed that pre-menopausal women differed in gut microbiome composition and more strongly from age-matched men than did post-menopausal women, indicating that sex differences were attenuated after menopause. Moreover, one of these studies,^73^ which used metagenomic sequencing, also found a greater number of differential KEGG pathways in younger adults (pre-menopausal women vs. younger men) than in older adults (post-menopausal women vs. older men), indicating that menopause may diminish sex-related differences in microbial functional potential. Using whole-metagenome shotgun sequencing data from 185 participants in South China (aged 14–74 y), Li et al.^81^ identified female-enriched metagenomic linkage groups assigned to *Bacteroides*, *Parabacteroides*, *Clostridium*, and *Akkermansia*. At the functional level, women showed enrichment of KEGG orthologs (KOs) involved in carbohydrate and energy metabolism, enzyme families, and glycan biosynthesis and metabolism, whereas men showed enrichment of KOs involved in nucleotide, cofactor, and vitamin metabolism.

**Figure 1.**
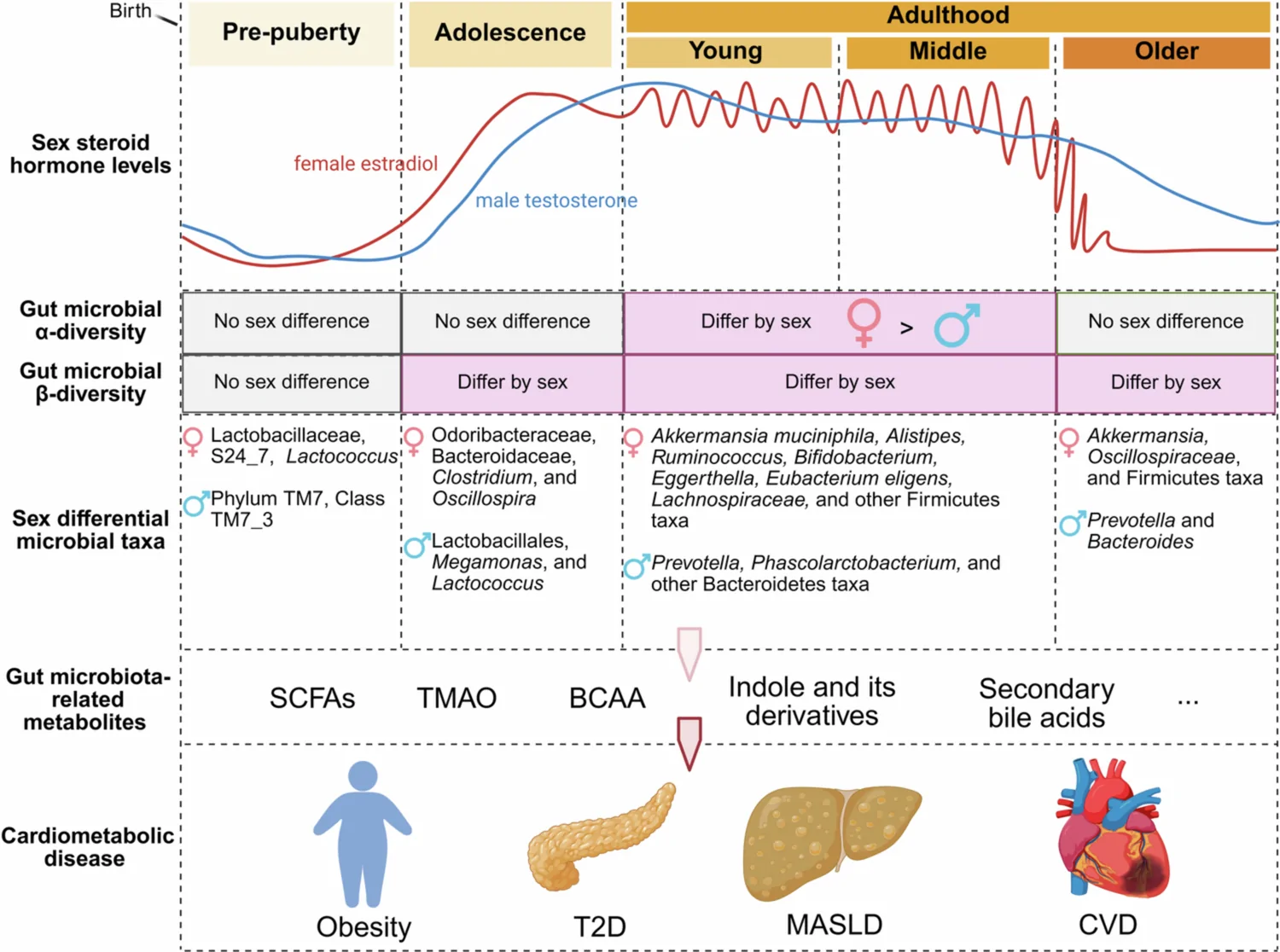
Summary of sex differences in gut microbiome features and sex steroid hormone trajectories across the life course. Schematic overview of age-related trajectories of female estradiol and male testosterone from birth through older adulthood. Both estradiol and testosterone rise during puberty, peak during the reproductive period, and decline with aging.^35^ ^,^ ^83^ ^,^ ^88^ Sex differences in gut microbial α-diversity, β-diversity, and differentially abundant taxa are summarized by life stage. Evidence for adulthood is primarily derived from large-scale studies with sample sizes exceeding 1000 participants (see Table 1), whereas findings for stages with more limited data availability (i.e., pre-puberty, adolescence, and older adulthood) reflect patterns consistently reported across existing studies. Sex-differential microbial taxa denote bacterial taxa enriched in females and males, respectively. The figure also shows representative gut microbiota-related metabolites and cardiometabolic diseases that were discussed in this review. This figure was created using BioRender.com. Abbreviations: SCFA, short-chain fatty acids; TMAO, trimethylamine N-oxide; BCAA, branched-chain amino acids; T2D, type 2 diabetes; MASLD, metabolic dysfunction-associated steatotic liver disease; CVD, cardiovascular disease.

In older adults, sex differences in the gut microbiome persist but tend to weaken with aging, likely reflecting converging hormone levels for women and men and increasing influence of non-sex-related factors. Some studies involving older adults observed significant β-diversity differences between women and men,^17^ ^,^ ^62^ ^,^ ^75^ but not α-diversity; another study^64^ reported no significant sex differences in β-diversity after menopause. Sex differences in gut microbiome features across the life course are summarized in Figure 1.

Although the specific differentially abundant taxa vary across studies, several taxa have been consistently identified across geographically distinct populations. Among younger and middle-aged adults (for women often in pre-menopausal stage), women tended to have higher abundances of taxa such as *A. muciniphila*,^70^ ^,^ ^74-76^ ^,^ ^81^ *Alistipes,* ^68^ ^,^ ^74^ ^,^ ^76^ ^,^ ^80^ *Ruminococcus,* ^66^ ^,^ ^69^ ^,^ ^73^ *Bifidobacterium*,^65,69^ *Eggerthella*,^69^ ^,^ ^70^ ^,^ ^74^ *Eubacterium eligens*,^70,76^ *Lachnospiraceae,* ^68^ ^,^ ^70^ ^,^ ^76^ and other Firmicutes taxa,^68^ ^,^ ^74^ ^,^ ^76^ ^,^ ^81^ whereas men often had higher enrichment in *Prevotella,* ^58^ ^,^ ^61^ ^,^ ^69^ ^,^ ^73^ ^,^ ^75^ ^,^ ^76^ *Phascolarctobacterium*,^65,76^ and other Bacteroidetes taxa.^62^ ^,^ ^73^ ^,^ ^79^ ^,^ ^82^ In contrast, the number and magnitude of sex-differential taxa were reduced among older adults (for women often in post-menopausal stage) compared with younger adults: women tended to have higher abundances of *Akkermansia*, Oscillospiraceae, and Firmicutes taxa, while men more often showed enrichment of *Prevotella* and *Bacteroides.* ^59^ ^,^ ^62^ ^,^ ^64^ ^,^ ^67^ ^,^ ^73^ ^,^ ^75^ Certain taxa appear to differ between women and men as adults irrespective of age, such as *Akkermansia and Prevotella*. It is worth noting that a recently published meta-analysis, the largest to date examining sex-associated gut microbiome features, analyzed data from 5505 individuals (3288 women and 2216 men; age > 16 y) across 13 countries from Asia, Africa, Europe, and North America.^89^ This study reported significantly higher α-diversity in women than in men, as well as significant sex differences in β-diversity in more than 80% of the included studies, as assessed by permutational multivariate analysis of variance. After adjustment for age, body mass index, and sequencing depth, the directions of sex-specific associations with the gut microbiome were broadly consistent across studies, with women exhibiting higher abundances of *A. muciniphila*, *Intestinimonas butyriciproducens*, *Bilophila wadsworthia*, *Gordonibacter pamelaeae*, *Ruthenibacterium lactatiformans*, and 2 MetaCyc pathways (e.g., L-lysine fermentation to acetate and butanoate), whereas men showed higher abundances of *Phascolarctobacterium succinatutens*, *Prevotella copri*, *Mitsuokella*, *Catenibacterium mitsuokai*, *Allisonella histaminiformans*, and 13 MetaCyc pathways (e.g., superpathway of L-aspartate and L-asparagine biosynthesis and petroselinate biosynthesis). Across five shotgun metagenomic studies^70^ ^,^ ^73-75^ ^,^ ^81^ (sample sizes ranged from 131 to 2338) and a recently published meta-analysis (5505 individuals),^89^ sex differences extended beyond microbial composition to microbial functional potential, although functional pathways were not consistent across studies. In general, women tended to show enrichment of functions related to SCFA production, sex steroid hormone metabolism, and carbohydrate and energy metabolism. In contrast, men tended to exhibit enrichment of pathways involved in nucleotide metabolism, cofactor and vitamin metabolism, amino acid biosynthesis, and bile acid transport. In addition, two studies^70^ ^,^ ^75^ identified sex differences in sulfur metabolism (e.g., sulfate transport, reduction, and assimilation). Menopause may attenuate sex-related differences in microbial functional potential; however, additional large-scale metagenomic studies are needed to obtain robust findings. Overall, the presence of sex differences in gut microbiome features during adulthood has been widely reported, while menopause and/or aging may act as potential modifiers attenuating microbial divergence between women and men. Longitudinal studies with repeated gut microbiome measurements should explicitly disentangle the effects of menopause and aging to capture sex-related microbial variation.

### GMRMs

Human studies examining sex differences in GMRMs remain limited. Large-scale efforts profiling the human metabolome do not examine sex differences, rather only considering sex as an adjustment or stratification factor in analysis.^90^ ^-^ ^92^ Most studies that do report sex differences in GMRMs have been conducted in disease-specific populations and rarely incorporate concurrent assessment of the gut microbiome, thereby constraining mechanistic interpretation. One early study by Li et al.^79^ investigated seven individuals from a Chinese family (age range: 1.5–95 y) using repeated fecal samples profiled by denaturing gradient gel electrophoresis and repeated urine samples analyzed by NMR spectroscopy. This study reported higher urinary creatine and lower 3-aminoisobutyrate levels in females, which were positively correlated with *Ruminococcus bromii* and *Bacteroides thetaiotaomicron*, respectively. In a Danish cohort of 205 adults, Cui et al.^93^ reported age-dependent sex differences in fecal metabolites: younger women had lower levels of valeric acid, isovaleric acid, butyric acid, and ethanol than younger men, whereas older women had lower valeric acid and propionic acid levels than older men. Several studies have focused exclusively on specific classes of GMRMs. *LPS.* In a study of 462 Spanish patients with coronary heart disease but without T2D (~60 y), plasma LPS levels were lower in women than in men.^94^ In addition, a previous study showed that men generally have stronger inflammatory responses to LPS compared to women. ^95^ ^,^ ^96^ *SCFAs*. Among 198 Italian patients with type 1 diabetes (19–79 y), women had higher serum acetic acid and total SCFA levels than men.^97^ Data from the Microbiome and Alzheimer's Risk Study (287 US adults, 13% with dementia; mean age 68 y) showed higher fecal SCFA abundances (e.g., acetate, propionate, butyrate, valerate) in women compared with men.^98^ *TMAO*. Blood TMAO levels were significantly higher in males compared to females among 592 healthy Finnish individuals at all six time points from age 11 to 26,^99^ 144 healthy Italian adults (mean age: 32 y),^100^ and the aforementioned Spanish study of coronary heart disease patients.^94^ Among 56 Australian patients with acute myocardial infarction, women exhibited significantly higher serum TMAO levels within the first day after AMI than men, with differences persisting until the beginning of cardiac rehabilitation and disappearing by the end of cardiac rehabilitation.^101^ *Indole and its derivatives.* No human study has explicitly examined sex differences as a primary focus, yet descriptive analyses by sex have suggested potential differences. A study of 318 Chinese adults (mean age: 50 y) reported that women were more likely to have higher serum indole-3-propionate levels than men.^102^ *Secondary bile acids.* Among 52 patients with nonalcoholic fatty liver disease (mean age: 49 y), women had higher serum levels of glycolithocholic acid, isolithocholic acid, and 12-ketolithocholic acid, as well as fecal lithocholic acid compared with men; in contrast, serum hyodeoxycholic acid, fecal 7-ketolithocholic acid, and ursodeoxycholic acid were significantly lower in women than men.^103^ Collectively, evidence for sex differences in GMRMs remains sparse, particularly in the general population. Existing studies are often limited by small sample sizes, population diversity, disease-specific study, lack of age-phase consideration, and targeted measurements of selected metabolites rather than comprehensive metabolomic profiling. These gaps also underscore the necessity for further large longitudinal studies incorporating multi-biospecimen sampling (e.g., fecal, blood, or urine) for untargeted metabolomics profiling with simultaneous assessment of gut microbiome, to clarify how sexual dimorphism in the gut microbiome influences microbial and host metabolism.

### Other gut microbiota (fungi, viruses, and archaea)

Emerging evidence suggests that sex may also influence the gut mycobiome, virome, and archaeome in human studies; however, findings remain limited and inconsistent across studies. (1) *Gut mycobiome*. In 111 healthy Italian participants (62 females and 49 males, mean age: 10 ± 8 y), culture-based analysis found more fungal isolates and species from females than males, while ITS1 sequencing in a subset of 57 participants showed sex differences in overall community composition and higher abundances of *Aspergillus* and an unidentified *Tremellomycetes* taxon in males than females;^104^ however, no sex differences in α-diversity were observed, which was consistent with another study in the Netherlands^105^ that compared the observed fungal amplicon sequence variant richness in 125 women and 38 men (mean age: 36 ± 12 y) using Internal Transcribed Spacer 2 (ITS2) sequencing. In 923 Polish adults (439 women and 484 men > 18 y) profiled by fecal whole-metagenome sequencing, they reported higher fungal evenness in women than men.^106^ A recent reanalysis of 1533 public ITS1 datasets identified *Acremonium* as associated with sex.^107^ Additional population-based evidence has been largely null: sex was not associated with human gut mycobiome variation in 217 Chinese adults from two regions,^108^ with fungal community patterns in 186 Slovenian adults,^68^ or with fungal enterotypes in a pooled analysis of 3363 samples from 16 cohorts.^109^ Collectively, these studies do not establish a consistent sex-specific gut fungal profile or demonstrate that fungal differences contribute to sex disparities in disease and health. Inconsistency may reflect low fungal biomass, marked temporal and interindividual variability, transient dietary or environmental fungi, limited characterization of hormonal status, and methodological differences among culture, ITS1 or ITS2 amplicon sequencing, and shotgun metagenomic sequencing. (2) *Gut virome*. A few recent large-scale studies suggest that sex is associated with selected viral community features. In 4198 Japanese participants (1730 women and 2468 men, mean age: 66 ± 13 y), sex was associated with 68 viral operational taxonomic units and 24 viral clusters.^110^ Men had higher abundances of phages predicted to infect *Prevotella* and *Megamonas*, whereas women had *Faecalibacterium*- and *Ruminococcus*-related phages. These patterns broadly paralleled sex differences in the corresponding bacterial hosts, suggesting that sexual dimorphism in the virome may partly reflect differences in the bacteriome. Furthermore, a pooled analysis of 8130 fecal metagenomes from infants younger than three years reported that gender significantly influenced the structure of the gut virome, although no consistent set of viruses enriched in girls or boys was established.^111^ More recently, analysis of viromes from 4261 Chinese participants (spanning childhood to older adulthood) showed that sex explained a small but significant proportion of overall virome variation.^112^ Women had greater viral richness and diversity than men, particularly between 30 and 49 y of age, while sex differences in specific viral families varied by age; for example, Microviridae was more abundant in men aged 30–39 y compared to women. These findings together suggest modest and age-dependent sex differences in the gut virome, but no reproducible sex-specific viral signature has yet emerged. Moreover, current studies predominantly characterize DNA bacteriophages recovered from bulk metagenomic data, with limited assessment of RNA viruses and eukaryotic viruses. (3) *Gut archaeome*. Archaea are commonly included in gut microbiome studies due to their possession of a 16S rRNA gene (captured in 16S rRNA amplicon sequencing) and presence in many databases used for shotgun metagenomic sequencing data. A global analysis of 1167 archaeal genomes recovered from human gastrointestinal metagenomes across 24 countries found that archaeal protein profiles could predict host sex with greater than 70% accuracy, with prediction improving when protein abundances were considered. This finding suggests that the taxonomic or functional composition of the archaeome may contain sex-associated features.^113^ In contrast, a metagenomic study of 792 Chinese adults suggested that gender was not associated with archaeome composition.^114^ Indirect evidence has also been obtained from breath methane, which reflects the metabolic activity of intestinal methanogenic archaea. An early study of 256 adults reported a higher prevalence of methane producers among women than men (49% vs. 33%),^115^ and a more recent study of 104 UK adults similarly found that women were more likely than men to be methane producers (38% vs. 25%).^116^ Overall, no female- or male-specific archaeal signature has been established. Importantly, given the connected nature of the gut microbiome, future studies should investigate sex differences in cross-kingdom interactions among bacteria, fungi, viruses, archaea, and other microbial communities.

## Mechanism of sex differences in gut microbiome and GMRMs

Intestinal microbiota transplant experiments in germ-free mice suggest that recipient sex plays a vital role in shaping the composition of the engrafted gut microbiota.^117^ Multiple observational human studies have also observed sex differences in the gut microbiome (and few in GMRMs), yet the underlying biological mechanisms remain incompletely understood. Sex hormones are recognized as a primary driver of these differences and provide a compelling framework for explaining sex-specific variation in microbial features across the life course. Existing evidence supports a sex-specific and bidirectional relationship between the host and the gut microbiota mediated by sex hormones.^118^ ^,^ ^119^ This section focuses on the mechanistic role of sex hormones in shaping the gut microbiome and its metabolic outputs, while also briefly summarizing other host and environmental factors that may contribute to sexual dimorphism of the gut microbiome.

**Sex hormones**. During early life (i.e., from birth to pre-puberty), sex does not appear to influence the gut microbiome composition. Deep sequencing of colonic contents in pre-pubescent mice reported no significant sex differences in bacterial community composition.^120^ Additional animal studies demonstrated that male and female mice have similar microbial communities after weaning, whereas sex differences develop at puberty and become more evident in adulthood.^121^ ^,^ ^122^ After puberty, female rodents have lower abundances of *Bacteroidetes* compared with males.^122^ These findings suggest that sex-related differences in the gut microbiome emerge after puberty, likely reflecting hormonal effects. Moreover, sex hormones can override the effects of sex chromosomes in shaping gut microbial composition;^123^ for example, *A. muciniphila* was identified as an estradiol-responsive species.

Trajectories of estradiol in females and testosterone in males across the human life course are summarized in Figure 1. The maturation of the gut microbiome parallels hormonal transitions across the lifespan.^83^ A shift towards sexual dimorphism of the gut microbiome is likely to emerge around puberty. In teenaged dizygotic twins (13–17 y), opposite-sex pairs exhibit greater microbial dissimilarity than same-sex pairs, but such differences were absent during infancy (1-12 months).^55^ Another study of children aged 5–15 y^57^ reported sex differences in gut microbial composition among pubertal children, but such differences were not observed before puberty likely due to gonadal quiescence and limited sex-specific development.^83^ Moreover, a longitudinal study of teenagers (at the age of 13 y)^124^ reported that the gut microbiome of girls transitioned toward a more adult-like profile as puberty progressed, suggesting a close interplay of sex hormones and gut microbiome during puberty. Together, evidence from both human and animal studies supports a prominent role of hormonal factors in shaping the gut microbiome from puberty onward.

**Figure 2.**
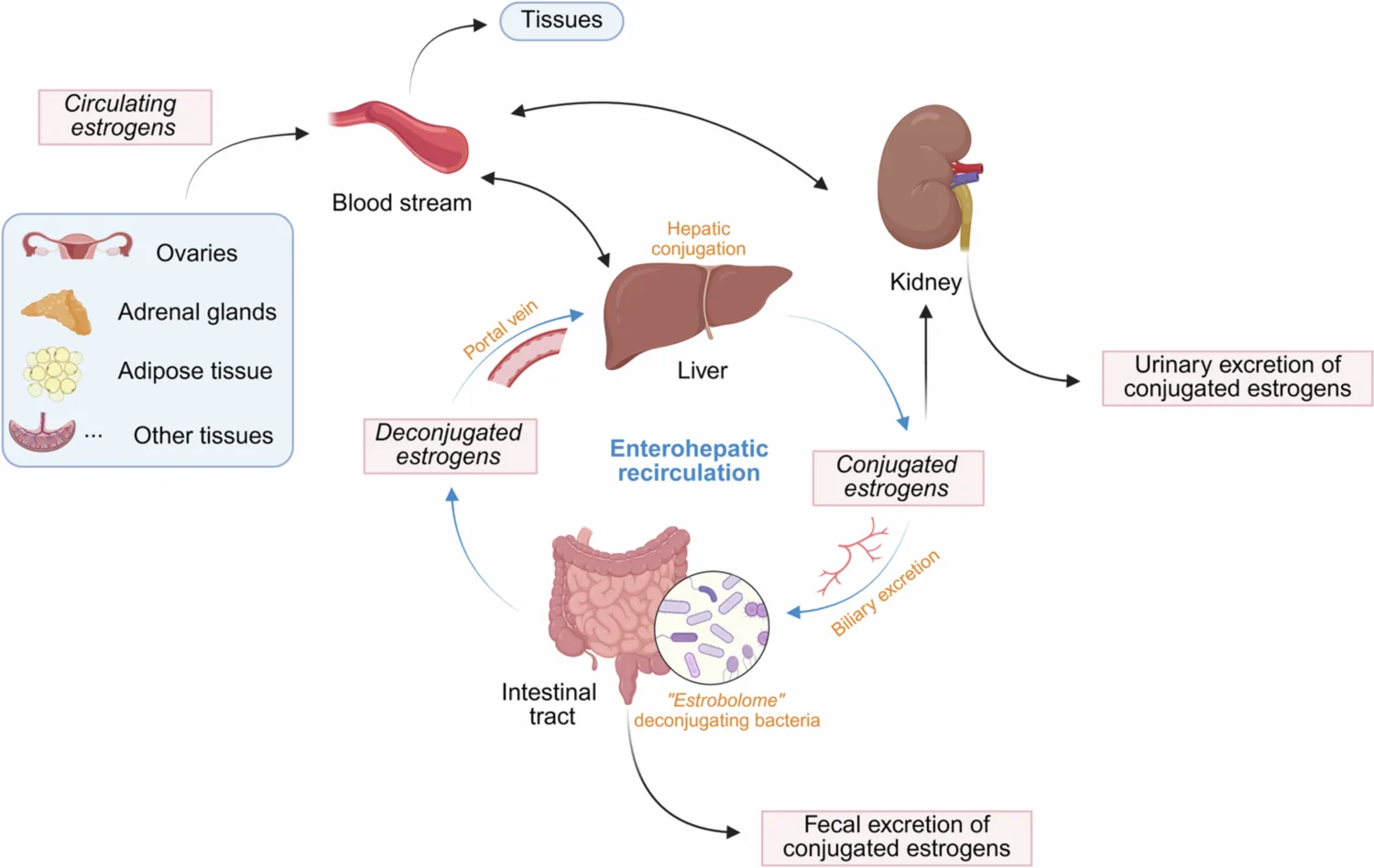
Enterohepatic recirculation of estrogens by the gut microbiota (estrobolome). Estrogens are primarily synthesized in the ovaries, adrenal glands, and adipose tissue, and circulate in the bloodstream in free or protein-bound forms to reach peripheral tissues. In the liver, estrogens undergo first-pass metabolism and are conjugated with glucuronide or sulfate groups, increasing their water solubility and facilitating elimination via urinary excretion or biliary secretion into the intestinal tract. Within the gut, conjugated estrogens can be directly excreted in feces or deconjugated by gut microbial enzymes, including β-glucuronidases and sulfatases, produced by estrogen-modulating bacterial communities collectively referred to as the *estrobolome*. Deconjugated (bioactive) estrogens may then be reabsorbed through the intestinal epithelium, return to the liver via the portal vein, and re-enter systemic circulation through enterohepatic recirculation. This figure was created using BioRender.com and adapted from Peters et al.^35^ and Kwa et al.^139^

Sex hormones and the gut microbiome interact bidirectionally, forming a biological basis for hormone-driven differences in microbial composition between women and men. Specifically, after production by the gonads, sex hormones are released into the bloodstream and metabolized by the liver, where they undergo hydroxylation followed by conjugation with glucuronide or sulfate groups. These conjugated forms are then excreted into bile and enter the intestinal tract. Within the intestine, certain microbes possess enzymes capable of deconjugating hormones, enabling their reabsorption into the enterohepatic circulation and subsequently reaching target cells, tissues, and organs.^35^ The collection of microbial genes involved in hormone deconjugation is termed the “estrobolome”.^125^ Bile-excreted hormones (such as estrogens, progesterone, and androgens) are subject to microbial deconjugation and recirculation. Figure 2 illustrates the enterohepatic recirculation of estrogens by the estrobolome. Through these mechanisms, sex hormones serve as substrates for microbial metabolism, while the gut microbiome simultaneously regulates circulating hormone levels, reinforcing their reciprocal influence. Beyond regulating microbial composition, sex hormones also impact adipose tissue distribution, energy and glucose homeostasis, and lipid metabolism differently for women and men, in which the gut microbiome may modify the metabolic effect of sex hormones.^3^ Most mechanistic evidence originates from animal models,^122^ ^,^ ^126-129^ in which a bidirectional relationship between gut microbiota and sex steroid hormones has been demonstrated: gut microbial enzymes (e.g., microbial β-glucuronidase) regulate hormone metabolism, and hormonal shifts, in turn, influence gut microbiota composition. Furthermore, sex differences in gut microbiota composition contribute to sexually dimorphic profiles of GMRMs.^130^ ^-^ ^133^ Sex-related variations in GMRMs are considered partly driven by sex steroid hormones. For example, testosterone inhibited expression of the hepatic TMAO-producing enzyme (flavin-containing monooxygenase 3, FMO3)^134^ and modulated bile acid signaling pathways,^135^ while estrogen can promote the growth of gut bacteria involved in SCFA production.^136^ In addition, increasing evidence suggests that sex hormone and gut microbiota interactions could contribute to sexually dimorphic susceptibility to metabolic diseases.^127^ ^,^ ^137^ ^,^ ^138^

Consistent with findings from experimental models, observational studies in humans have also reported associations between circulating sex steroid hormones and gut microbiome composition. Sex steroid hormones, mainly estrogen and testosterone, are considered prominent regulators of sexual dimorphism in gut microbiota.^140^ A systematic review^141^ showed that, among healthy women, higher circulating estrogen levels were associated with greater microbial diversity, increased abundance of Bacteroidetes, and lower abundance of Firmicutes and the Ruminococcaceae family. In healthy men, higher testosterone levels were positively associated with increased microbial diversity and higher abundances of *Ruminococcus* and *Acinetobacter*. Our recent study examined the associations of 14 serum sex hormones and sex hormone-binding globulin (SHBG) with the gut microbiome in men with and without HIV, and identified numerous microbial species associated with adrenal precursors, androgens, estrogens, and SHBG.^142^ Sex hormones and their fluctuations throughout the life course influence the gut microbiome composition accordingly, and their associations should be interpreted in the context of age and medical conditions.

**Other factors**. Although sex hormones are the most well-characterized drivers of sexual dimorphism in the gut microbiome, immune function represents another important mechanism underlying sex-specific microbial patterns. The intestinal tract is recognized as one of the body's largest immune organs, playing a pivotal role in immune regulation and host defense.^143^ Similar to the gut microbiome, the immune system undergoes sexual differentiation during puberty and remains sexually dimorphic throughout adulthood,^144^ ^-^ ^146^ with women generally exhibiting stronger innate and adaptive immune responses than men.^147^ Both innate^148^ and adaptive^149^ immune responses regulate the gut microbiome, while the gut microbiome in turn affects mucosal immune system development and function.^150^ The interactions between gut microbiome and immune system impact host homeostasis and disease.^150^ Animal studies also show that sex differences in immune responses are associated with sex differences in microbiome composition.^151^ ^,^ ^152^ Therefore, sex-specific differences in the immune system may contribute to distinct gut microbiome profiles in women and men.

In addition, sex differences in gut microbiota may reflect underlying differences in gut physiology and different responses to environmental factors, such as sex-related behaviors (e.g., diet,^153^ physical activity,^154^ smoking,^155^) medications,^70^ disease,^156^ and sociocultural factors.^157^ GMRMs can reflect sex-specific gut microbial responses to dietary interventions and may serve as functional readouts of the gut microbiome. An animal study demonstrated that feeding a high-fat diet induces sex-dependent alterations in gut microbial composition and colonic bacterial metabolites (short/medium-chain fatty acids).^158^ Similarly, another study has shown that male and female rats exhibit distinct fecal SCFA profiles in response to the same oligofructose-containing diet.^159^

It is worth noting that most studies were limited by analytical approaches that treat sex as a stratification variable rather than a primary exposure of interest and did not exclusively investigate the underlying pathways between sex and gut microbiome. Future studies should move beyond stratified analyses to incorporate mechanistic frameworks.

## Sexually dimorphic gut microbiota and GMRMs in CMD

Accumulating evidence has demonstrated that gut microbiome and GMRMs may influence the initiation and progression of CMD;^160^ ^-^ ^162^ however, there remains a great lack of information on sex-related differences. Most human studies have evaluated sex differences in microbial features or associations between microbial features and CMD separately, rather than testing the complete pathway from sex or hormonal status through microbial functions and metabolites to disease development. Identifying potential microbial mechanisms underlying sex-specific CMD risk is therefore essential to develop effective prevention and therapeutic strategies, especially given increasing interest in personalized medicine for women and men. In this section, we summarize current evidence on the roles of sexually dimorphic gut microbiota and GMRMs in CMD.

**Obesity**. Obesity is a major risk factor for CMD.^163^ Sex differences in obesity are well-established and reflect complex interactions among biological, hormonal, and environmental factors. Globally, obesity is more prevalent in women than in men^13^ and marked sex differences also exist in fat distribution.^164^ Men with obesity typically have more visceral adipose tissue (“apple shape” of central fat distribution), whereas women more commonly accumulate subcutaneous adipose tissue (“pear shape” of peripheral fat distribution).^165^ ^,^ ^166^ Despite well-established sex differences in adiposity, evidence linking the gut microbiome to body fat distribution is limited and largely indirect.^167^ ^,^ ^168^ Firmicutes and Bacteroidetes are the two dominant bacterial phyla in the human gut, making up > 90% of the total bacterial species.^20^ Therefore, shifts in the relative abundances of Firmicutes and Bacteroidetes may reflect changes in host health and disease status. Increased F/B ratio, a marker of gut dysbiosis, has been associated with fat accumulation,^169^ ^-^ ^171^ whereas a lower F/B ratio has been linked to weight loss.^169^ ^,^ ^172^ Several studies have reported sex differences in the F/B ratio,^64^ ^,^ ^82^ with women exhibiting a higher F/B ratio than men; however, evidence suggests that these differences may be modified by adiposity, as higher F/B ratio has been observed among women with obesity than men with obesity.^64^ Furthermore, diet is closely related to both the gut microbiome and obesity. Increased F/B ratio represents a greater capacity to harvest energy from the diet,^173^ and thus is associated with obesity. Animal studies further support the role of sexually dimorphic gut microbiota in modulating diet-induced obesity. For example, when fed the same high-fat diet, male mice gained significantly more weight and showed a greater increase in the F/B ratio than female mice, while female mice had higher fecal acetate levels, a SCFA associated with improved metabolic flexibility and energy regulation.^174^ These observations suggest that females may be more resistant to diet-induced obesity, partly due to sex-specific gut microbiota and the production of related metabolites. Moreover, sex differences in fat distribution have been related to differences in sex hormone levels. Sex hormones may metabolically interact with the gut microbiome to influence obesity risk and fat distribution. Estrogen plays a role in regulating adipose tissue deposition,^175^ and the decline in estrogen levels after menopause is associated with loss of subcutaneous fat and an increased risk of abdominal obesity in women.^176^ A mouse study^138^ demonstrated that gut microbiota interact with the loss of female sex hormones to exacerbate metabolic dysfunction, including weight gain and hepatic lipid accumulation, which highlighted a synergistic effect of hormonal and microbial changes. Collectively, these findings indicate that sexually dimorphic gut microbiota and GMRMs may provide biological plausibility for sex-specific susceptibility to obesity; however, direct evidence on whether they mediate sex-specific obesity risk or body fat distribution remains limited. When evaluating the association between gut microbiome and obesity and/or body fat distribution, sex along with age and diet should be explicitly considered to better capture sex-specific microbial patterns that underlie obesity risk.

**T2D**. Epidemiological evidence suggests that T2D is more prevalent in men than in women, particularly during middle adulthood, whereas post-menopausal women and men tend to be more insulin resistant compared with pre-menopausal women.^177^ Both clinical and experimental studies support that sex steroid hormones largely contribute to sexual dimorphism in energy balance and metabolic homeostasis in the post-pubertal stage, which are the main determinants of sex differences in T2D susceptibility.^178^ ^,^ ^179^ Estrogens protect women from T2D during the reproductive period, while low androgen levels contribute to the development of T2D in men; conversely, androgens predispose women to insulin resistance and T2D.^180^ Human studies have reported higher enrichment of *Bacteroides* and *Prevotella* taxa in men than women.^58^ ^,^ ^59^ ^,^ ^75^ Notably, *Prevotella copri* and *Bacteroides vulgatus* were identified as major contributors to BCAA biosynthesis, which is associated with a greater risk of insulin resistance.^181^ Sex differences in BCAA and related catabolites have been consistently reported, and men had higher circulating BCAA levels than metabolically comparable women.^182^ In terms of sex steroid hormones, estrogen deficiency may modulate insulin resistance and pancreatic β-cell workload by reducing beneficial bacteria (e.g., *A. muciniphila*, *Bifidobacterium* spp., and *Lactobacillus* spp.) while increasing potentially pathogenic taxa within *Bacteroides*.^183^ Activation of hepatic estrogen receptor-α (ER-α) appears protective against T2D and is associated with decreased Firmicutes and increased Bacteroidetes abundance.^184^ A mouse study reported that sexual differences in glucose metabolism disappeared when gut microbiota were depleted, highlighting the critical role of the microbiome in mediating sex-specific glucose metabolism.^185^ Mechanistically, androgens can impair glucose homeostasis in part by gut microbiota and circulating glutamine and glutamine/glutamate ratio (i.e., GMRMs), thereby affecting insulin sensitivity and driving sex differences in glucose metabolism. Collectively, these findings identify plausible interactions among sex steroid hormones, microbial functions, and amino acid metabolism in the regulation of glucose metabolism. However, direct evidence in humans that these pathways explain sex differences in T2D risk remains limited, and most evidence derives from observational studies or animal models.

**MASLD**. Formerly termed non-alcoholic fatty liver disease (NAFLD), MASLD is the most prevalent chronic liver disease worldwide and a leading cause of liver-related morbidity and mortality.^186^ Pronounced sex differences have been reported in MASLD epidemiology: MASLD is more common in men than pre-menopausal women, whereas its prevalence significantly increases in women after menopause and becomes comparable to men.^186^ These patterns suggest a protective role of sex steroid hormones, particularly estrogen, which has been shown to attenuate hepatic lipid accumulation by enhancing fatty acid oxidation and improving insulin sensitivity.^187^ In contrast, higher testosterone levels have been shown to contribute to the progression and severity of MASLD in women, whereas low testosterone levels are associated with increased MASLD risk in men,^188^ underscoring sex-specific hormonal influences on liver metabolism. The gut–liver axis, defined by the bidirectional interaction between the gut microbiota and the liver, is important in MASLD pathogenesis.^189^ Through the portal vein, the liver is constantly exposed to GMRMs, which collectively influence hepatic lipid metabolism, inflammation, and immune responses.^186^ Relatively few studies have examined whether gut microbial alterations differentially contribute to MASLD development in women and men, highlighting an important area for future investigation.^190^ ^,^ ^191^ For example, women with MASLD had a lower abundance of *Christensenella* and higher abundance of *Limosilactobacillus*, whereas men had lower *Beduinibacterium* and higher *Anaerotruncus* abundance, suggesting distinct microbial contributions to MASLD between sexes.^190^ In a high-fat diet rat model,^158^ male rats had higher abundances of Firmicutes and *Bilophila*, which are associated with impaired intestinal barrier function, endotoxemia, and hepatic steatosis; in contrast, female rats had increased levels of propionate, a SCFA with protective effects on obesity, insulin resistance, and hepatic lipid accumulation. Consistent with these microbial and metabolic differences, female rats developed less severe liver inflammation and steatosis than male rats when they were fed a high-fat diet. Beyond microbial composition, emerging evidence links sex-dimorphic gut microbiota and bile acid profiles to different immune responses in the liver, which further affects hepatic lipid metabolism.^192^ In addition, estrogen decline in post-menopausal women was associated with gut dysbiosis and alterations in GMRMs, including reduced SCFA production, impaired antimicrobial peptide expression, and disrupted lipid metabolism, which together may contribute to MASLD development.^193^ In support of this notion, a female rat model demonstrated that gut microbiota alterations following bilateral oophorectomy led to abnormal fatty acid biosynthesis, particularly in linoleic acid metabolism, which may impact the development of hepatic steatosis.^194^ Taken together, these findings suggest that sex-specific interactions between gut microbiota, GMRMs, and sex hormones can critically shape MASLD development and progression. Thus, future basic and clinical studies should emphasize the importance of incorporating sex, hormonal status, and age for MASLD management.

**CVD**. CVD encompasses a spectrum of disorders affecting the heart and blood vessels, such as coronary artery disease, stroke, and heart failure. Although the overall lifetime risk of CVD is similar between women and men, CVD is diagnosed at a younger age in men, whereas CVD risk rises sharply in women after menopause.^195^ The post-menopausal increase in CVD risk has been largely attributed to estrogen decline, which promotes dyslipidemia, insulin resistance, systemic inflammation, and a redistribution of body fat toward a more visceral pattern.^196^ ^,^ ^197^ The gut-heart axis describes the bidirectional communication between the gastrointestinal tract and the heart, which underscores the critical role of gut microbiome and GMRMs in CVD pathogenesis.^198^ ^,^ ^199^ Firmicutes, *Ruminococcus, Eubacterium, Eggerthella,* and *Akkermansia* have been shown to be more abundant in the gut microbiome of women compared to men.^82^ ^,^ ^200^ Many Firmicutes species are major producers of SCFAs,^201^ particularly butyrate (e.g., *Roseburia* and *Faecalibacterium prausnitzii*), which exert cardioprotective influence by reducing blood pressure, improving insulin sensitivity, and exerting anti-inflammatory and antioxidant properties.^201^ ^-^ ^203^ Beyond SCFA production, some Firmicutes harbor genes encoding microbial β-glucuronidase,^204^ ^,^ ^205^ a key enzyme involved in estrogen deconjugation and enterohepatic recycling.^128^ Through this mechanism, the gut microbiota can modulate circulating estrogen levels, thereby indirectly influencing lipid metabolism and vascular function. In parallel, Firmicutes are major contributors to the production of TMAO,^206^ a pro-atherogenic and pro-thrombotic GMRM strongly linked to CVD risk.^207^ Men generally have higher levels of circulating TMAO than women,^99^ ^,^ ^100^ and such sex difference may be partially explained by estrogen-mediated suppression of hepatic FMO3, a key enzyme in the conversion of trimethylamine (TMA) to TMAO, through estrogen receptor-α (ERα) signaling.^208^ ^,^ ^209^ Consequently, higher estrogen levels in pre-menopausal women may limit TMAO generation and confer vascular protection. Sex differences are also observed in bile acid metabolism in relation to cardiovascular health. Microbial taxa such as *Eubacterium*, *Ruminococcus*, and *Eggerthella* catalyze the conversion of primary bile acids to secondary bile acids, the majority of which are reabsorbed and recirculated to the liver.^210^ ^-^ ^212^ In addition, antibiotic-induced reductions in *Firmicutes* abundance, accompanied by decreased levels of secondary bile acids, have been linked to impaired insulin sensitivity in patients with metabolic syndrome.^213^ Women tend to have higher circulating levels of gut-derived secondary bile acids compared to men. ^130^ These metabolites activate nuclear receptors such as the farnesoid X receptor (FXR), which plays a protective role in CVD by regulating bile acid synthesis, lipid metabolism, and glucose homeostasis.^214^ *A. muciniphila*, a mucin-degrading bacterium that is more frequently enriched in women,^215^ has emerged as a particularly important cardioprotective taxon. *A. muciniphila* can reduce local inflammation of the vascular endothelium, increase SCFA secretion and indole-3-propionic acid production, regulate the renin-angiotensin system, and protect against arterial calcification and atherosclerosis.^216^ ^-^ ^218^ Importantly, gut microbiota also interact with major hormonal transitions across the lifespan that shape CVD risk, particularly the menopausal decline in estrogen and the accompanying pro-atherogenic shift in lipid profiles in women.^219^ ^,^ ^220^ Moreover, gut microbiome alterations may be both a cause and a consequence of key CVD risk factors, including dyslipidemia, dysglycemia, hypertension, and obesity, forming complex bidirectional feedback loops.^153^ In addition, evidence supporting sex-specific microbial mechanisms in CVD remains limited in human studies, and prospective studies designed to evaluate sex-related microbial pathways are needed. Identification of certain sex-specific gut microbial species, GMRMs, and their functional contributions to CVD could generate a curated database of candidate microbes, facilitating the development of microbiome-informed strategies for CVD risk assessment and intervention.

## Research gaps and future directions

Although substantial evidence supports the presence of sex differences in gut microbiome composition, the specific taxa identified differ considerably across the existing studies (Table 1), with sample sizes varying widely, from 7 to 9381 participants. These discrepancies are likely attributable to various influencing factors, such as age, diet, geographic location, race/ethnicity, body composition, and comorbid medical conditions. Moreover, the majority of human studies relied on 16S rRNA gene sequencing, which limits taxonomic resolution and functional inference; whereas only five studies^70^ ^,^ ^73-75^ ^,^ ^81^ have used shotgun metagenomic sequencing so far, and the small number of such studies may hinder further insights into sex-specific microbial functions and metabolic capacity. A growing number of 16S rRNA gene sequencing and shotgun metagenomic datasets generated for diverse research purposes have been deposited in publicly accessible repositories.^221^ ^-^ ^225^ Although these datasets were not originally designed to study sex differences, those containing sufficiently detailed metadata (e.g., sex, age, race/ethnicity, and other relevant variables) could be reanalyzed to characterize sex-associated variation in the gut microbiome. These resources represent an important yet underutilized opportunity to conduct harmonized analyses across diverse populations, identify both shared and population-specific sex-associated microbial taxa and functional pathways, and examine whether these associations vary across racial/ethnic backgrounds and life stages. Future studies leveraging these public resources, together with standardized analytical pipelines and metadata harmonization, will increase statistical power, improve the reproducibility and generalizability of findings, and further advance our understanding of sex differences in gut microbial composition and function.

Age is a major determinant of gut microbial composition, yet its interaction with biological sex remains insufficiently characterized. While several studies have compared gut microbiome differences across life stages (e.g., pre-puberty vs. puberty, or pre- vs. post-menopause), no prospective longitudinal studies have tracked within-person changes in the gut microbiome across the life course in a sex-specific manner. Single time-point microbiome assessments preclude disentangling sex effects from aging effects and limit causal inference. Applying new study designs (e.g., propensity score matching) and longitudinal studies spanning hormonal transitions across the lifespan are therefore essential to determine whether sex differences in gut microbiome composition and function emerge, accelerate, or attenuate at specific life stages.

The gut microbiota primarily exerts its functions through the production of GMRMs. Despite growing recognition that GMRMs serve as key mechanistic links between the gut microbiome and host health,^226^ ^,^ ^227^ very few human studies have simultaneously examined sex differences in gut microbiome composition and microbial metabolites within the same population. Existing studies have focused primarily on a limited set of commonly reported metabolites, such as SCFAs, TMAO, BCAA, and secondary bile acids. Moreover, while GMRMs can be measured in fecal, blood or urine samples, it remains unclear how metabolite levels across different sample types reflect gut microbial activity and their biological relevance. Future studies should integrate multi-matrix untargeted metabolomic profiling (e.g., fecal, plasma, urine) to better characterize how sexually dimorphic microbial functions interact with host physiology.

Further, a critical unanswered question is the extent to which sex differences in CMD are mediated by sexually dimorphic gut microbiota and GMRMs. While CMD share overlapping risk factors and etiologic pathways, the specific roles of sex-specific microbial taxa and metabolites in shared or disease-specific pathways remain largely unexplored. Future research should systematically evaluate how sexually dimorphic gut microbiota and GMRMs contribute to the development of interconnected CMDs, by identifying common or distinct microbial-metabolic mechanisms.

The biological mechanisms underlying sex differences in gut microbial alterations are largely attributed to sex steroid hormones; however, the interactions remain incompletely understood. Sex hormones fluctuate dynamically across the life course and may exert stage-specific and sex-specific effects on both the gut microbiome and cardiometabolic health. Yet sex hormone measurements are not routinely incorporated into population-based studies. This gap is particularly pronounced in women of reproductive age, where hormonal variability across the menstrual cycle poses additional challenges to hormone measurement. An additional critical gap in the current literature is the paucity of studies examining the gut microbiome and GMRMs among pregnant women, a life stage characterized by profound and dynamic hormonal, metabolic, and immunological changes. Pregnant women are frequently excluded from microbiome studies (i.e., few studies compare pregnant women with age-matched non-pregnant women) or are analyzed without accounting for gestational stage or hormonal milieu. Similarly, given the comparatively greater focus on menopause, estrogen, and the gut microbiome in women, longitudinal investigations spanning young, middle, and older adulthood are necessary to determine whether age-related declines in androgens contribute to shifts in gut microbial composition, GMRMs, and cardiometabolic risk in men. Beyond sex hormones, other contributors, such as immune function, lifestyle behaviors, and sociocultural factors, are increasingly recognized as potential drivers of sexually dimorphic gut microbiota and may jointly contribute to sex disparities in CMD risk. To advance the field, large longitudinal studies are needed to integrate gut microbiome with metabolomics profiling and major influencing factors (e.g., sex hormones, biomarkers of immune function, lifestyles). Such an integrative approach will be essential to elucidate the specific mechanistic pathways through which sex differences in the gut microbiome and GMRMs arise and to determine how these microbial pathways influence CMD development, as well as other sex-biased diseases.

Finally, it remains unclear whether modifying the gut microbiota and GMRMs can be leveraged therapeutically to reduce sex-specific CMD risk, as most existing studies are observational. Well-designed randomized clinical trials targeting the gut microbiome, including personalized dietary interventions, probiotics, or modulation of microbial functional enzymes and GMRMs, could explicitly evaluate sex-specific responses to CMD prevention and therapies. Accounting for sex differences in microbiome composition, function, and host-microbe interactions may be critical for optimizing prevention and treatment strategies for CMD, as well as informing future basic, clinical, and translational research. Incorporating sex hormone measurements in both women and men will be essential to disentangle hormonal effects from chronological aging and to elucidate hormone-microbiome interactions underlying sex-specific CMD susceptibility.

## Conclusions

In summary, sex differences in the gut microbiome and GMRMs are dynamic across the life course and may contribute to CMD (Figure 1). Overall, sex-related differences in gut microbial composition are minimal before puberty, emerge with pubertal hormonal changes, are most pronounced during reproductive adulthood, and attenuate with aging and menopause, in parallel with longitudinal alterations in sex steroid hormones. During early and middle adulthood, women (particularly pre-menopausal women) generally have a more favorable gut microbial profile than men, characterized by higher microbial diversity and enrichment of taxa with cardioprotective properties, such as *A. muciniphila* and species from *Eubacterium*, *Bifidobacterium*, and Firmicutes. These compositional differences are accompanied by sex-specific microbial metabolites, such as BCAA, SCFAs, secondary bile acids, and TMAO, which may influence host glucose homeostasis, lipid metabolism, immune response, and vascular function. In contrast, men are more likely to have greater abundances of the *Prevotella* and *Bacteroides* and higher levels of GMRMs associated with insulin resistance and atherosclerosis. Menopause represents a critical transition point, given that estrogen decline is associated with reduced gut microbial diversity, diminished estrobolome capacity, unfavorable alterations in GMRMs, and increasing similarity of gut microbiome composition between post-menopausal women and men. These microbial and metabolic shifts may contribute to an increased risk of CMD in women after menopause. Current evidence suggests that sex differences in gut microbiome and GMRMs contribute to sex-specific CMD susceptibility. Incorporating sex, age, and hormonal status into microbiome research is essential for advancing mechanistic understanding and developing sex-informed microbiome-based strategies for CMD prevention and treatment, which is of great significance to clinical research and practice.

## Supplementary Material

re_supplementary_table_1.docx re_supplementary_table_1.docx
