# Supplementary material for: Sex differences in the gut microbiome and related metabolites: role in cardiometabolic disease
Source: Gut Microbes. 2026 Aug 26;18(1):2721752. doi: 10.1080/19490976.2026.2721752 (PMC13523995; doi:10.1080/19490976.2026.2721752)
Supplement: Supplementary file 1 [file KGMI_A_2721752_SM3212.docx]

**Supplementary Table 1:** Summary of the literature search strategy for human studies included in this narrative review.

| **Database** | **Representative search terms** | **Scope** |
| --- | --- | --- |
| PubMed | *1. Sex differences in the gut microbiome*  (“gut microbiome” OR “gut microbiota” OR “gut bacteria”) AND (“sex differences” OR sex OR gender)  *2. Sex differences in gut microbiota-related metabolites*  (“gut microbiota-related metabolites” OR “gut microbiota-derived metabolites” OR “gut microbial metabolites”) AND ("sex differences" OR sex OR gender)  *3. Sex differences in gut microbiome and cardiometabolic disease*  #1 AND (“cardiometabolic disease” OR obesity OR “type 2 diabetes” OR “metabolic dysfunction-associated steatotic liver disease” OR “cardiovascular disease”)  *4. Sex differences in gut microbiota-related metabolites and cardiometabolic disease*  #2 AND (“cardiometabolic disease” OR obesity OR “type 2 diabetes” OR “metabolic dysfunction-associated steatotic liver disease” OR “cardiovascular disease”) | Epidemiologic studies |
| Web of Science | The same as above | Epidemiologic studies |
| Google Scholar | The same as above | Additional recent epidemiologic studies |
